# [Expression of Concern] Regulation of NADPH oxidase (Nox2) by lipid rafts in breast carcinoma cells

**DOI:** 10.3892/ijo.2025.5814

**Published:** 2025-10-23

**Authors:** Rama Rao Malla, Hari Raghu, Jasti S. Rao

Int J Oncol 37: 1483-1493, 2010; DOI: 10.3892/ijo_00000801

Following the publication of the above paper, a potential problem regarding the presentation of the co-localization experiments shown in Fig. 5A and C was brought to the Editor's attention by a concerned reader. Specifically, the Flotillin/gp91 co-localization panels (Fig. 5A) appeared to be unexpectedly similar to the Flotillin/p22 panels (Fig. 5C), even though, according to the Materials and methods section, the different antibody treatments that were reported might have precluded the possibility of these images looking so similar.

The authors were contacted by the Editorial Office to offer an explanation for this potential anomaly in the presentation of the data in this paper (or to clarify how the experiments had been performed), although up to this time, no response from them has been forthcoming. Owing to the fact that the Editorial Office has been made aware of potential issues surrounding the scientific integrity of this paper, we are issuing an Expression of Concern to notify readers of this potential problem while the Editorial Office continues to investigate this matter further.

